# The assessment of glutathione, glutathione peroxidase, glutathione reductase, and oxidized glutathione in patients with periodontitis—A systematic review and meta‐analysis

**DOI:** 10.1002/cre2.907

**Published:** 2024-06-16

**Authors:** Khadijah Mohideen, Chandrasekaran Krithika, T. Jeyanthikumari, N. V. Vani, Safal Dhungel, Snehashish Ghosh

**Affiliations:** ^1^ Department of Oral Pathology and Microbiology, Sathyabama Dental College and Hospital Sathyabama Institute of Science and Technology Chennai India; ^2^ Meenakshi Academy of Higher Education and Research Chennai India; ^3^ Tamilnadu Government Dental College Chennai India; ^4^ Department of Epidemiology Cancer Institute (WIA) Chennai India; ^5^ Department of Oral and Maxillofacial Surgery College of Medical Sciences Bharatpur Nepal; ^6^ Department of Oral Pathology College of Medical Sciences Bharatpur Nepal

**Keywords:** antioxidants, glutathione peroxidase, glutathione reductase, periodontitis

## Abstract

**Objective:**

The present systematic review explored the involvement of enzymatic and nonenzymatic antioxidants in periodontitis, drawing from established literature.

**Materials and Methods:**

The research approach encompassed an extensive electronic search from 2000 to 2023 across databases such as PubMed, Science Direct, and Wiley Online Library and cross‐referencing using specific keywords.

**Results:**

The initial literature exploration generated a total of 766 articles. After thoroughly examining the abstracts, 693 articles were excluded from consideration due to duplication and lack of relevance to the central research inquiry. Following that, 73 articles were left for in‐depth evaluation. Following a qualitative assessment, 35 studies that satisfied the inclusion criteria were chosen, while 38 were removed for not meeting the necessary standards. Within this selection, a meta‐analysis was conducted on 11 articles that provided consistent data for quantitative synthesis. Specifically, the analysis of glutathione (GSH) levels in serum samples revealed a standardized mean difference (SMD) of −5.552 µg/mL (CI 95%: −9.078 to −2.026; P‐0.002). In contrast, the analysis of glutathione peroxidase (GPx) enzymes in gingival crevicular fluid (GCF) samples displayed an overall SMD of 2.918 ng/µL (CI 95%: 0.372–5.465; P‐0.025), while salivary samples exhibited an overall SMD value of 0.709 U/l (95% CI: −1.907–3.325; P‐0.596) which is of insignificant.

**Conclusion:**

The systematic review findings suggest a notable decrease in antioxidant enzymes across various systemic biological samples among patients with periodontitis, contrasting with the results from gingival tissue samples meta‐analysis of GPx enzyme.

## 1 INTRODUCTION

Periodontitis, a prevalent inflammatory disease, arises from bacterial infection and is exacerbated by an aberrant host response. Its consequences are far‐reaching, as it not only leads to the degradation of crucial tooth‐supporting tissues but also exerts systemic health influences. The pathogenesis of periodontitis involves a cascade of events triggered by bacterial infection, followed by an unbalanced host response, resulting in tissue damage and destruction. Reactive oxygen species (ROS), primarily generated by hyperactive neutrophils, play a pivotal role in this process. However, the antioxidant defense system often fails to counterbalance the overproduction of ROS, further contributing to oxidative stress and tissue damage. This damage is characterized by increased levels of metabolites associated with lipid peroxidation, DNA damage, and protein impairment. Moreover, the local and systemic antioxidant activities are disrupted, leading to heightened oxidative stress in periodontitis (Wang et al., 2017).

The nonenzymatic antioxidant GSH stands for reduced glutathione, while glutathione disulfide (GSSG) represents its oxidized counterpart. Regrettably, GSSG is sometimes labeled as oxidized glutathione (GSH), although its accurate designation is glutathione disulfide. The prevailing predominance of GSH over GSSG, with a ratio as high as 98%, significantly influences cellular redox potential (Averill‐Bates, 2023). Notably, GSH actively detoxifies reactive oxygen species, contributing to overall cellular health by reducing equivalents for many biochemical pathways (Jones et al., 2011). GSH can also be subject to oxidation through interactions with radicals, leading to the creation of glutathione disulfide (Kalyanaraman et al., 1996).

Instances of oxidative stress elevate GSSG levels, prompting an activation of the glutathione reductase enzyme. The enzymatic antioxidant glutathione reductase (GR) operates as a homodimer, with each monomer housing a single FAD molecule. Its primary function involves facilitating the conversion of GSSG into GSH. This activation results in the restoration of glutathione by utilizing reducing equivalents derived from NADPH (Åslund et al., 1997). Another enzymatic antioxidant, glutathione peroxidase (GPx), facilitates hydrogen peroxide detoxification. The GPx enzyme is a selenoprotein that efficiently reduces peroxides of hydrogen and lipids to water and lipid alcohols by coupled oxidation of GSH to GSSG. The considerable GSH‐to‐GSSG ratio is pivotal in maintaining the intracellular redox equilibrium, detoxifying reactive oxygen species, and orchestrating the glutathione redox cycle (Valko et al., 2007). The present article delves into the status of vital enzymes like reduced GSH, oxidized glutathione GSSG, GR, and GPx in various biological samples of patients with periodontitis. By investigating these mechanisms, the present paper sheds light on the critical relationship between cellular health, oxidative balance, and the physiological implications of periodontitis.

## 2 MATERIALS AND METHODS

### 2.1 Focused research question

The PICO question focuses on oxidant‐antioxidant status (GSH, GSSG, GR, GPx) in patients with and without periodontitis.

Is there a discernible difference in the oxidant‐antioxidant status in the periodontitis group in comparison to the healthy control group?

### 2.2 Electronic search process

To identify previously published articles that explored antioxidant levels in periodontitis, with a specific focus on GSH, GR, GSSG, and GPx levels from 2000 to 2023, all in the English language, we initiated a literature search across electronic databases, including PubMed, Science Direct, and Wiley Online Library. Cross‐referencing was also employed to expand our search scope.

In the PubMed database, we conducted searches using the below‐mentioned keywords in the title or abstract: (1) Antioxidant enzymes; Glutathione Reductase; Glutathione; Glutathione Disulfide; Glutathione Peroxidase; connected by the Boolean operator OR. (2) keywords: Periodontitis connected with AND. Filters were applied to the articles retrieved in the PubMed database searches to include only those categorized as “Abstract, Clinical Trial, Comparative Study, Evaluation Study, Observational Study,” involving humans, published in English, and spanning from January 1, 2000, to June 30, 2023.

In the Science Direct database, we utilized the keywords “antioxidant enzymes” and “periodontitis.” Articles identified in the Science Direct database were filtered based on publication date (2000–2023) and article type (research articles). Subject areas included Biochemistry, Genetics and Molecular Biology, Medicine and Dentistry, Pharmacology, Toxicology, and Pharmaceutical Science.

Finally, the employed title or abstract keywords in the Wiley Online Library database were: “Glutathione reductase OR Glutathione OR Glutathione peroxidase OR antioxidant enzymes AND periodontitis.” Articles identified in Wiley Online Library searches were restricted to those published in English between 2000 and 2023.

### 2.3 Screening for suitability

The articles' titles and abstracts were assessed to determine their pertinence and identify duplication.

### 2.4 Inclusion criteria

1. The studies involved human adults as study subjects.
2. The articles under consideration must include healthy control (C) and periodontitis (P) groups.
3. The control group consists of periodontally and systemically healthy individuals.
4. The periodontitis group should not have undergone prior periodontal therapy recently.
5. The studies referenced relevant periodontal parameters such as probing pocket depth, clinical attachment level, or bleeding on probing within the periodontitis cohort.
6. The studies explored at least one of the antioxidant markers, including GSH, GR, GSSG, or GPx, in patients diagnosed with periodontitis.
7. Research encompassing systemic diseases associated with periodontitis is included if it incorporates a separate assessment group for the specified marker evaluation, comprising both systemically healthy controls and a periodontitis group.
8. Studies involving individuals who smoke and have periodontitis are considered, provided they include non‐smokers in both the periodontitis and control groups as distinct evaluation categories.
9. Research evaluating the specified marker's post‐therapy effects in periodontitis is included if they furnish baseline values for both the periodontitis and control groups.

### 2.5 Exclusion criteria

1. The duplicated studies are published in different journals but stem from the same subjects and authors.
2. The studies have involved subjects with smokers or pregnant females without involving a control group.
3. The studies have involved diabetes patients or those with systemic diseases without involving the control group.
4. Case reports, letters, and literature reviews
5. Studies that do not provide adequate information for comparison with other studies.
6. Studies dealt with unmatched aims for the evaluation of antioxidative status.
7. Studies utilized markers other than the selected antioxidant enzymes for the evaluation.
8. Studies displayed the results solely in histogram representation and did not provide sufficient data for comparison of antioxidant enzymes.
9. Observational studies involving only children.

#### 2.5.1 Literature research

Initially, two authors conducted independent searches within electronic databases and meticulously extracted pertinent studies based on a comprehensive review of titles, abstracts, and complete texts. The identified articles were evaluated based on predefined inclusion and exclusion criteria. Any reports that did not meet these criteria were excluded from the refined selection. Throughout this procedure, any differences in judgment were subject to thorough discussion with a third author, resulting in a resolution. Moreover, reference lists from existing studies were scrutinized to detect any additional articles that the initial search strategy might not have uncovered.

#### 2.5.2 Evaluation of the articles

Two evaluators independently assessed studies using New Castle‐Ottawa criteria. These criteria included: 1) Study Group Selection: Examining how well the cases were defined and representative of the population. 2) Comparison with Control Group: Considering factors like smoking and systemic diseases that impact antioxidant status in the control group comparison. 3) Exposure Assessment: This involved ethics approval, investigator blindness, similarity in selected groups, conflicts of interest statement, and nonresponse rate.

#### 2.5.3 Data collection

Two researchers independently extracted the data from retrieved articles manually. The information gathered from the full‐text assessment encompassed author names, publication year, country of origin, criteria defining periodontitis and control groups, age demographics information, sample sizes, and the methodology employed for assessing GSH, GSSG, GR, and GPx. The corresponding marker values for patients with periodontitis and control groups were recorded, including mean and standard deviation or median (range), relevant units, and statistical significance indicators. The data collection did not extend to seeking information the original study investigators did not provide. The extracted details underwent a validation process, during which a third author reviewed and confirmed the accuracy of the collated information. Any disparities were deliberated upon to achieve a consensus and ensure the precision of the compiled data.

#### 2.5.4 Statistical analyses

Meta‐analyses were carried out when three or more studies reported antioxidant marker measurements consistently, either as mean with standard deviation (SD) or median with range, within the same sample. Due to the variation in methodology and units employed across the chosen studies for assessing antioxidant enzyme levels, the meta‐analysis utilized a random effects model with the standardized mean difference (SMD) and a 95% confidence interval (CI) as the summary statistic to evaluate variations in enzyme levels. Studies that did not provide SD values or contained values outside the acceptable range were excluded from the meta‐analysis. The medium to high heterogeneity was indicated by an I² value exceeding 50%. The statistical investigation was accomplished using the software comprehensive meta‐analysis (Biostat) version 3.

## 3 RESULTS

This meta‐analysis included all relevant cross‐sectional and case‐control studies in the literature to provide a comprehensive quantitative summary of the available evidence. The PubMed search yielded 38 articles, the Science Direct search resulted in 526 articles, the Wiley Online Library search produced 189 articles, and 13 additional articles were identified through cross‐referencing. Following a thorough screening of the 766 articles, 693 were excluded due to duplication and lack of relevance to the research focus. After excluding these articles, the full‐text versions were retrieved from the initial 73 papers with similar objectives to the current systematic review (SR). After careful evaluation, we excluded 38 articles due to incompatible data. Ultimately, 35 articles were selected based on the SR's exclusion and inclusion criteria.

**Figure 1:**
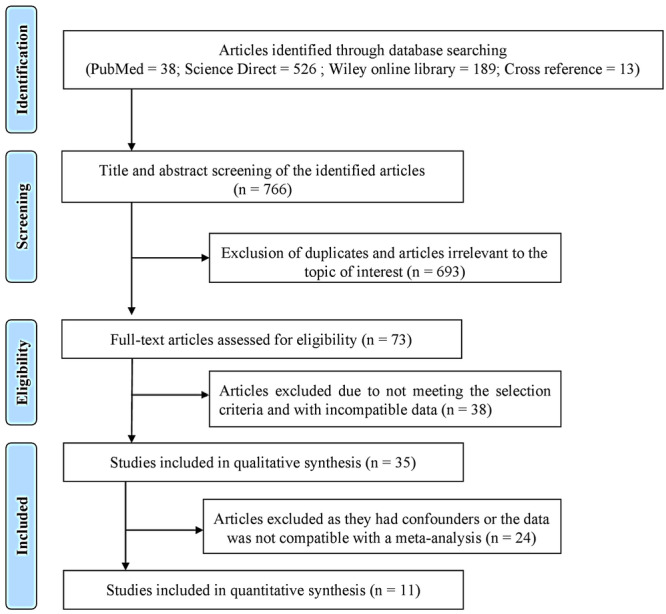
Flowchart for the process of selection of the studies.

Furthermore, clinical interventional studies that provided baseline pre‐therapy data were included. When an individual study had more than two study groups, this systematic review specifically focused on systemically healthy periodontitis and the healthy group. Notably, the studies selected by the investigators for this systematic review demonstrated strong inter‐examiner reliability agreement, with a κ value of 0.81. Finally, 11 articles contained compatible data for meta‐analysis. The process for selecting these studies is described in Figure 1, while the results of the quality assessment scale can be found in Table 1. Each item within the assessment was assigned a score of one point, and ratings were distributed on a scale of 0–9. Studies falling within the range of 0–2 were categorized as poor quality, 3–5 as medium quality, and 6–9 as high quality. All the studies included in this review were assessed as medium or high quality.

**Table 1:** New Castle Ottawa quality scores for the included articles.

	Selection				Comparability		Exposure				
Study details	Case criteria	Case representability	Control selection	Control criteria	Matched known confounding factors	Matched impending confounding factors	Confidentiality ‐ patient records	Examiners blindness	Similarity between the groups	Non‐responsiveness	Total score
Almerich Silla et al. (2015)	_+_	+	+	+	+	×	+	×	×	×	6
Aziz et al. (2013)	+	+	+	+	+	+	+	×	+	×	8
Borges Jr. et al. (2007)	+	+	+	+	+	+	+	×	+	×	8
Canacki et al. (2009)	_+_	+	+	+	+	+	+	×	+	×	8
Chapple, (2002)	+	+	+	+	+	+	+	×	+	×	8
Dhotre et al. (2012)	+	+	+	+	×	+	+	×	+	×	7
Emekli Alturfan et al. (2013)	×	+	+	×	+	×	+	+	×	×	5
Gharbi et al. (2019)	+	+	+	+	+	+	+	×	+	×	8
Grant et al. (2010)	+	+	+	+	×	+	+	×	+	+	8
Guentsch et al. (2008)	_+_	+	+	+	+	+	+	×	+	+	9
Hendek et al. (2015)	+	+	+	+	+	+	+	×	×	×	7
Kluknavska et al. (2020)	+	+	+	×	×	×	+	×	+	×	5
Kluknavska et al. (2021)	+	+	+	+	×	×	+	×	+	×	6
Arunachalam et al. (2019)	+	+	+	+	+	+	+	×	+	×	8
Martu et al. (2016)	+	+	+	+	+	+	+	×	+	×	8
Miricescu et al. (2014)	+	+	+	+	+	+	+	×	+	×	8
Novakovic et al. (2013)	+	+	+	+	+	+	+	×	+	×	8
Panjamurthy et al. (2005)	+	+	+	+	+	+	+	×	×	×	7
Patel et al. (2009)	_+_	+	+	+	+	+	+	×	+	×	8
Patel et al. (2012)	+	+	+	+	+	+	+	×	+	×	8
Praveen Dahiya et al. (2016)	+	+	+	+	+	+	+	×	×	×	7
Punj et al. (2017)	+	+	+	+	+	+	+	×	×	×	7
Senouci et al. (2021)	+	+	+	+	+	+	+	×	+	×	8
Shankarram et al. (2015)	×	+	+	+	×	+	+	×	×	×	5
Smitha et al. (2017)	+	+	+	+	+	×	+	×	×	×	6
Sreeram et al. (2015)	+	+	+	+	+	+	+	×	+	×	8
Thomas et al. (2013)	+	+	+	+	+	+	+	×	+	×	8
Thomas et al. (2021)	×	+	+	+	+	+	×	×	+	×	6
Tonguc et al. (2011)	+	+	+	+	+	+	+	×	+	×	8
Trivedi et al. (2014)	_+_	+	+	+	+	+	+	×	+	×	8
Trivedi et al. (2015)	+	+	+	+	+	+	+	×	+	×	8
Tsai et al. (2005)	_+_	_+_	+	+	×	+	+	×	+	×	7
Verghese et al. (2023)	+	+	+	+	+	+	+	+	×	+	9
Wei et al. (2004)	+	+	+	+	+	+	+	×	+	×	8
Zuidan (2009)	×	+	+	+	+	×	×	×	+	×	5

### 3.1 Explanations for exclusion of studies after full‐text evaluation

Following a thorough assessment of the complete texts, 38 articles did not meet the selection criteria. Among these, three studies presented histogram representations for assessing antioxidant enzymes (Monea et al., 2014; Oktay et al., 2015; Sree & Sethupathy, 2014). Two studies focused on pregnant women with periodontitis (Canakci et al., 2007; Gümüş et al., 2015). Furthermore, three studies were excluded because they lacked control group details (Arana et al., 2017; Garg et al., 2006; Sree & Sethupathy, 2014). Another study targeted the Aggressive Periodontitis group alone (Arunachalam et al., 2019).

#### 3.1.1 Data summary

The compiled data and assessment methodologies from the identified studies for assessment of GSH, GR, GSSG, and GPx were presented in a designated format, as outlined in Tables 2, 3, 4, 5, respectively. The Cohen's κ value for inter‐examiner reliability was computed at 0.81, signifying a significant consensus between the examiners.

**Table 2:** The assessment of GSH in various biological samples between the Periodontitis and control groups in the selected studies.

Reference	Country	Study design	Sub‐group	Age Range or mean ± SD P/C	Periodontitis criteria	Unit of measurement	P – (Mean ± SD or median with range	P ‐ Sample number (M/F)	C ‐ (Mean ± SD or median with range	C ‐Sample number (M/F)	Result	P value	Method
Borges Jr. et al. (2007)	Brazil	CC	Gingival tissue	52.9 ± 5.0/51.1 ± 9.6	BOP‐ 5 to 6 sites; CAL ≥ 3 mm; PPD ≥ 5 mm; radiographic bone loss	μmol g^−1^	0.46 ± 0.06	9 (4/5)	0.38 ± 0.08	9 (4/5)	Insignificantly increased	0.459	Beutler (1975)
Chapple, (2002)	UK	CS	GCF	46.1/46.9	Gustafsson & Asman (1996)	µM	1183.1 ± 580.3	10 (5/5)	1899.8 ± 494.4	10 (5/5)	Significantly decreased	<0.222	HPLC Analysis
Emekli‐ Alturfan et al. (2013)	Turkey	CC	Saliva	26–59 (both)	NA	mg/dl	0.69 ± 0.04	7	0.38 ± 0.04	12	Significantly increased	0.0001	Beutler (1975)
Grant et al. (2010)	UK	CC	GCF	43.6 ± 2.1/44.3 ± 2.3	PPD ≥ 5 mm; BOP; radiographic bone loss ≥30% of root length	mM	1182 ± 711	20 (8/12)	1953 ± 566	20 (8/12)	Significantly decreased	<0.001	Chapple, (2002)
Kluknavska et al. (2020)	Slovak Republic	CCCC	Saliva	NA	PPD ≥ 6 mm; bone resorption	nmol SH/mg prot	0.2834 (0.0189‐0.5980)	16	0.1652 ± (0.084‐0.749)	13	Significantly increased	<0.05	Floreani et al. (1997)
			Plasma				0.4322 (0.2057‐1.4117)	15	0.3536 ± (0.2206‐1.263)	12			
Kluknavska et al. (2021)	Slovak Republic	CC	Saliva	NA	PPD ≥ 6 mm; bone resorption; values 3,4 in CPITN	nmol SH/mg prot	0.289 (0.071‐2.058)	23	0.285 ± (0.053‐0.539)	43	Significantly increased	<0.001	Floreani et al. (1997)
Panjamurthy et al. (2005)	India	CC	Plasma	25–35 (both)	PPD > 3.5; Gingival recession (grade3); furcation involvement; tooth mobility grading	mg/dl	29.59 ± 3.68	25	43.63 ± 5.04	25	Significantly decreased	0.001	Beutler and Kelly (1963)
			Erythrocyte			mg/dl	30.97 ± 5.02		47.67 ± 3.52				
			Gingival tissue			n moles/mg protein	10.9 ± 1.23		6.32 ± 0.72		Significantly increased	0.001	
Senouci et al. (2021)	Algeria	CC	Saliva	24.06 ± 6.09/24.73 ± 1.38	Stage 3,4: grade C periodontitis	μmol/mL	35.06 ± 13.65	29 (6/23)	45.86 ± 11.48	28 (7/21)	Significantly decreased	<0.001	Weckbecker and Cory (1988)
Thomas et al. (2013)	India	Comparative study	Serum	30–60 (both)	CAL > 4 mm in 30% of the sites	µg/ml	56.93 ± 6.874	50	90.36 ± 6.564	50	Significantly decreased	≤0.005	Spectrophotometry method
Thomas et al. (2021)	India	RCT	Serum	30–60 (both)	NA	µg/ml	64.4014 ± 13.4374	100	86.58 ± 12.29954	100	Significantly decreased	<0.05	DTNB
Tsai et al. (2005)	Taiwan	CC	Saliva	NA	PPD ≥ 3 mm; PAL ≥ 2 mm; GI > 1	µM	373.04 ± 287.42	13	606.67 ± 191.02	9	Significantly decreased	<0.05	Spectrophotometric assay kit
Verghese et al. (2023)	India	LS	Serum	30‐60 (both)	Minimum 20 natural teeth; PPD at least 30% of sites; CAL > 3 mm; bone loss	µg/ml	13.6 ± 0.99	25	25.67 ± 1.29	25	Significantly decreased	<0.001	DTNB
Zuidan (2009)	Iraq	CC	Erythrocyte	22–60 (both)	NA	mg/dl	68.29 ± 16.42	50 (20/30)	76.61 ± 151.5	50 (23/27)	Significantly decreased	<0.05	Beutler and Kelly (1963)

Abbreviations: BOP, Bleeding on Probing; C, Control; CAL, Clinical Attachment Loss; CC, Case‐Control; CS, Cross‐sectional; GSH, Reduced Glutathione/Glutathione; LS, Longitudinal Study; P, Periodontitis; PPD, Periodontal Probing Depth; RCT ‐ Randomized Control Trial.

**Table 3:** The assessment of GR in various biological samples between the P group and control group in the selected studies.

Study name	Country	Study design	Sub‐group	Age range or mean ± SD P/C	Periodontitis criteria	Unit of measurement	P – (Mean ± SD or median with range	P ‐ sample number (M/F)	C‐Mean ± SD or median with range	C ‐ sample number (M/F)	Result	P value	Method
Borges Jr. et al. (2007)	Brazil	CC	Gingival tissue	52.9 ± 5.0/51.1 ± 9.6	BOP‐at least 5 to 6 sites; CAL ≥ 3 mm; PPD ≥ 5 mm; radiographic bone loss	μmol min^−1^ g^−1^	0.22 ± 0.06	9 (4/5)	0.28 ± 0.04	9 (4/5)	Insignificantly decreased	0.481	Carlberg & Mannervik (1985)
Gharbi et al. (2019)	North Africa	CC	Plasma	20–60 (both)	Jack and Caton (1999)	nmol/min/ml	28.55 ± 10.79	80 (33/47)	48.72 ± 8.02	50 (25/25)	Significantly decreased	0.001	Colorimetric assays
Kluknavska et al. (2020)	Slovak Republic	CC	Saliva	NA	PPD ≥ 6 mm; bone resorption	μkat/mg prot	0.8602 (0.7373–1.1311)	16	0.7407 (0.161–2.114)	13	Insignificantly increased	>0.05	GR, EC 1.8.1.7)Sigma‐Aldrich, Germany
			Plasma				0.9619 (0.5459–1.6588)	15	1.1015 (0.313–1.958)	12	Insignificantly decreased		
Kluknavska et al. (2021)	Slovak Republic	CC	Saliva	NA	PPD ≥ 6 mm; bone resorption; values 3,4 in CPITN	μkat/mg prot	0.695 (0.022–2.759)	23	1.098 (0.061–6.118)	43	Significantly decreased	<0.001	
Trivedi et al. (2014)	INDIA	CS and CC	Saliva	20–65 (both)	Armitage (1999)	U/min/mg protein	13.63 ± 6.46	30	24.57 ± 7.97	30	Significantly decreased	<0.001	Hazelton and Lang (1980)
			Blood			U/min/mg protein	10.22 ± 5.59		18.74 ± 5.25				
Trivedi et al. (2015)	India	CS and CC	Saliva	25–45 (both)	Armitage (1999)	U/min/mg protein	12.51 ± 6.39	30 (14/16)	25.5 ± 9.11	30 (15/15)	Significantly decreased	<0.001	Spectrophotometry

Abbreviations: BOP, Bleeding on Probing; C, Control; CAL, Clinical Attachment Loss; CC, Case‐Control; CS, Cross‐sectional; GR, Glutathione Reductase; P, Periodontitis; PPD, Periodontal Probing Depth.

**Table 4:** The assessment of GSSG in various biological samples between the P and control groups in the selected studies.

Study name	Country	Study design	Sub‐group	Age range or mean ± SD P/C	Periodontitis criteria	Unit of measurement	P ‐ mean ± SD or median with range	Sample number (M/F)	C‐ mean ± SD or median with range	Sample number(M/F)	Result	P value	Method
Borges Jr. et al. (2007)	Brazil	CC	Gingival tissue	52.9 ± 5.0/51.1 ± 9.6	BOP‐ at least 5 to 6 sites; CAL ≥ 3 mm; PPD ≥ 5 mm; radiographic bone loss	μmol g^−1^	0.17 ± 0.04	9 (4/5)	0.06 ± 0.01	9 (4/5)	Significantly increased	0.019	Beutler (1975); Tietze (1969)
Chapple, (2002)	UK	CS	GCF	46.1/46.9	Gustafsson & Asman 1996 [50]	µM	150.1 ± 44.9	10 (5/5)	256.8 ± 152.4	10 (5/5)	Significantly decreased	<0.026	HPLC
Grant et al. (2010)	UK	CC	GCF	NA	PPD ≥ 5 mm; BOP; radiographic bone loss ≥ 30% of root length	mM	157 ± 48	20 (8/12)	237 ± 123	20 (8/12)	Significantly decreased	<0.05	Chapple, (2002)

Abbreviations: BOP, Bleeding on Probing; C, Control; CAL, Clinical Attachment Loss; CC, Case‐Control; CS, Cross‐sectional; GSSG, Oxidized Glutathione; P, Periodontitis; PPD, Periodontal Probing Depth.

**Table 5:** The assessment of GPx in various biological samples between the P and control groups in the selected studies.

Study	Country	Study design	Sample type	Age range or mean ± SD P/C	Periodontitis criteria	Unit of measurement	P mean ± SD or median with range	Sample number (M/F)	C ‐mean ± SD or median with range	Sample number (M/F)	Result	P value	Method
Almerich‐Silla et al. (2015)	Spain	CC	Saliva	41–45/38–43	In at least 4 zones CAL ≥ 2 mm; PPD ≥ 5 mm	U/l	95.58 (93.12–98.04)	33 (19/14)	75.04 (72.35–77.72)	37 (15/22)	Significantly increased	<0.05	ELISA Kit
Aziz et al. (2013)	India	CC	Serum	37–50/39.64 ± 5.04	Armitage (1999)	U/g Hb	8.45 ± 1.2	134 (M)	14.3 ± 1.2	64 (M)	Significantly decreased	<0.001	Paglia and Valentine (1967)
Borges Jr. et al. (2007)	Brasil	CC	Gingival Tissue	52.9 ± 5.0/51.1 ± 9.6	BOP, at least 5 or 6 sites, CAL ≥ 3 mm, PPD ≥ 5 mm, radiographic bone loss	µmol/min/g	2.09 ± 0.34	9 (4/5)	0.8 ± 0.11	9 (4/5)	Significantly increased	0.006	Flohé and Günzler (1984)
Canacki et al. (2009)	Turkey	CS	Saliva	45.3 ± 0.97/42.7 ± 12.4	Armitage (1999)	U/l	74.2 ± 26.96	30 (15/15)	90.8 ± 23.62	30 (15/15)	Significantly decreased	<0.05	Paglia and Valentine (1967)
Dahiya et al. (2016)	India	CC	Serum	20–50(Both groups)	Armitage (1999)	µg/mgHb/min	2.8 ± 0.29	20 (10/10)	5.6 ± 0.215	20 (10/10)	Significantly decreased	<0.01	Rotruck et al. (1973)
Dhotre et al. (2012)	India	CC	Serum	NA	CAL ≥ 4 mm; PPD ≥ 4 mm with BOP	U/ml	1.9 ± 0.175	50	4.61 ± 0.26	25	Significantly decreased	<0.001	Ransel Kit
Guentsch et al. (2008)	Germany	PS	Saliva	46.3 ± 13.1/34.1 ± 11.8	at least 30% of teeth with pocket depth >5 mm	U/l	18.59 ± 15.995	30 (14/16)	6.75 ± 3.235	30 (14/16)	Significantly increased	<0.05	Paglia and Valentine (1967)
Hendek et al. (2015)	Turkey	PS	Serum	44.0 ± 15.0/33.0 ± 8.0	Armitage (1999)	U/ml	33.545 ± 15.665	47 (24/23)	41.84 ± 22.795	46 (23/23)	Insignificantly decreased	>0.05	ELISA Kit
			GCF			U/µl	8.275 ± 2.305		5.88 ± 1.58		Significantly increased	<0.05	
			Saliva			U/ml	33.7 ± 12.11		26.79 ± 14.87		Significantly increased	<0.05	
Kluknavska et al. (2020)	Slovak Republic	CC	Plasma	NA	PPD up to 6 mm and alveolar bone resorption with concomitant gingivitis	μkat/mg prot	1.0965 (0.1170–3.9474)	1516	0.2485 (0.0146– 4.1228)	1213	Insignificantly increased	>0.05	Sigma‐AldrichKit
			Saliva			μkat/mg prot	0.519 (0.0439–1.7105)		0.874 (0.2743– 7.4479)		Insignificantly decreased	>0.05	
Kluknavska et al. (2021)	Slovak Republic	CC	Saliva	NA	PPD up to 6 mm and alveolar bone resorption	μkat/mg prot	1.203 (0.033–5.342)	23	2.917 (0.041–6.809)	43	Significantly decreased	<0.001	Sigma‐AldrichKit
Arunachalam et al. (2019)	India	Comparative study	Saliva	20–60 (Both groups)	Flemmig (1999)	U/l	117.9 ± 5.765	60 (60/0)	147.57 ± 5.54	30 (30/0)	Significantly decreased	0.0001	Photometric RX Daytonaplus (Randox)
Martu et al. (2016)	Romania	CC	GCF	24–55 (Both groups)	2 sites with gingivitis and PPD of ≥4 mm	U/ml	736.75	16	742.8	15	Insignificantly decreased	>0.05	Paglia and Valentine (1967)
Miricescu et al. (2014)	Romania	CC	Saliva	51.26 ± 7.4/18.66 ± 2	At least 6 regions with PPD ≥ 4 mm, alveolar bone loss > 30% along with gingivitis	U/mg albumin	15.81 ± 7.22	20 (14/11)	28.16 ± 11.95	20 (20/5)	Significantly decreased	<0.05	GPxAssay Kit
Novakovic et al. (2013)	Serbia	PS	Saliva	39.2 ± 11.5/35.2 ± 7.1	Evidenced radiographic bone loss >30%, at least one periodontal pocket; PPD > 5 mm per quadrant with BOP	IU/l	1842.95 ± 157.76	21 (14/7)	1869.43 ± 185.31	21 (14/7)	Insignificantly decreased	>0.05	UV method, Ransel kit
Panjamurthy et al. (2005)	India	CC	Plasma	25–35 (Both groups)	PPD (>3.5 mm), grade III gingival recession, furcation involvement, and mobile teeth.	U/l	239.99 ± 38.53	25 M	197.32 ± 21.33	25 M	Significantly increased	<0.001	Rotruck et al. (1973)
			RBC			U/g Hb	38.2 ± 5.2		29.2 ± 1.9				
			Gingival tissue			U/g protein	20.2 ± 1.91		14.3 ± 1.22				
Patel et al. (2009)	India	CC	GCF	35.25 ± 3.39/32.80 ± 2.89	GI > 1; PPD ≥ 4 mm in 30% of sites; CAL ≥ 1 in 30% of sites; radiographic evidence of bone loss	ng/ml	30.89 ± 4.93	20	15.32 ± 3.06	20	Significantly increased	<0.05	Biotin and Steptavidin ‐ELISA Kit
Patel et al. (2012)	India	Intervention	GCF	35.10 ± 2.51/35.10 ± 2.02	GI >1 and CAL ≥1 mm in 30% of sites, alveolar bone loss with PPD ≥4 mm in 30% of sites	ng/µl	29.89 ± 4.03	10 (5/5)	14.01 ± 2.86	10 (5/5)	Significantly increased	<0.05	ELISA Kit
			Serum			ng/ml	103.43 ± 7.163		78.26 ± 4.649				
Punj et al. (2017)	India	Comparative study	Saliva	25–65 (Both groups)	Armitage (2004)	*μ*g/g protein	0.38 ± 0.09	20	0.34 ± 0.06	20	Significantly increased	<0.001	Rotruck et al. (1973)
			Serum			U/mg Hb	156.2 ± 84.4		167.7 ± 56.21		Significantly decreased	0.006	
Shankarram et al. (2015)	India	CC	Saliva	NA	NA	U/l	80.99 (76.83–85.15)	25	75.04 (72.35–77.72)	25	Significantly increased	<0.05	ELISA Kit
Smitha et al. (2017)	India	CC	Saliva	30–55/15–35	PPD ≥4 mm; LOA ≥3 mm	mg/dl	3.903 ± 0.80229	20	1.149 ± 0.2970	20	Significantly increased	<0.0005	Paglia and Valentine (1967)
			Gingival tissue			µg/g	43.25 ± 8.117		30.85 ± 7.562		Significantly increased		
Sreeram et al. (2015)	India	CC	Blood	41.0 ± 12.2/34.2 ± 12.0	BOP in >30% of sites, with PPD of 1‐3 mm, BOP and CAL ≥3 mm >30% of all quadrants	U/ml	3.68 ± 0.45	150 (114/36)	4.82 ± 0.24	150 (120/30)	Significantly decreased	<0.05	Paglia and Valentine (1967)
Tsai et al. (2005)	Taiwan	CC	Saliva	NA	PPD ≥3 mm; CAL ≥2 mm and GI ≥1	mU/ml	92.99 ± 74.4	13	92.9 ± 58.58	9	Insignificantly increased	>0.05	SpectroPhotometricAssay Kit
			GCF			µM	151.9 ± 77.7		50.66 ± 37.22		Significantly increased	<0.005	
Tonguc et al. (2011)	Turkey	CS	Gingival Tissue	20–50/25– 49	Armitage (1999)	U/mg protein	154.83 (110.83–220.65)	65 (32/33)	284.4 (237.3–421.4)	20 (11/9)	Significantly decreased		Flohé and Günzler (1984)ELISA Kit
			Blood			U/mg hemoglobin	14.7 (3.43‐43.86)		7.4 (3.4–18.6)		Significantly increased	<0.01	
Wei et al. (2004)	Taiwan	CS	GCF	45.1 ± 10/26.6 ± 1.9	GI >0; PPD >3 mm; LA > 3 mm	ng/µl	35.7 ± 35.89	19 (11.8)	17.21 ± 12.67	8 (3/5)	Significantly increased	<0.05	ELISA (R & D Systems Inc.)

Abbrevaitions: BOP, Bleeding on Probing; C, Control; CAL, Clinical loss of Attachment; CC, Case Control; CS, Cross‐sectional; GI, Gingival Index; GPx, Glutathione Peroxidase; LOA, Loss of Attachment; P, Periodontitis; PPD, Periodontal Pocket Depth.

### 3.2 Characteristics of studies involved in the SR

Most studies selected the participant's age group between 25 and 60 years. Periodontitis characterization predominantly relied on clinical indicators and radiological assessments such as bone loss. Ten studies provided the criteria reference articles for periodontitis (Armitage, 2004; Armitage,^1999^; Flemmig, 1999; Gustafsson & Åsman, 1996; Jack & Caton, 1999). The assessment method of the selected antioxidant enzymes is mentioned in Tables 2, 3, 4, 5.

Among the included studies, six incorporated smokers and non‐smokers with periodontitis (Borges Jr. et al., 2007; Dhotre et al., 2012; Guentsch et al., 2008; Hendek et al., 2015; Naresh et al., 2019; Tonguç et al., 2011). Concerning periodontitis diagnosis, three studies included generalized and aggressive periodontitis groups (AgP) for evaluation (Kluknavská et al., 2020; Kluknavská et al., 2021; Martu et al., 2016). Seven studies have included gingivitis as a separate evaluation group (Almerich‐Silla et al., 2015; Kluknavská et al., 2020; Kluknavská et al., 2021; Patel et al., 2009; Patel et al., 2012; Shankarram et al., 2015; Wei et al., 2004). One study differentiated between periodontitis, healthy control groups, and systemic conditions of ischemic heart disease (Punj et al., 2017). Additionally, four studies contrasted periodontitis groups with and without diabetes mellitus (Thomas et al., 2013; Thomas et al., 2021; Trivedi et al., 2014; Verghese et al., 2023). Furthermore, five studies assessed therapeutic outcomes and compared baseline pretreatment values of the periodontitis and control groups (Grant et al., 2010; Guentsch et al., 2008; Novaković et al., 2013; Tsai et al., 2005; Verghese et al., 2023).

### 3.3 Assessment of antioxidant markers in the included studies

The current systematic review (SR) comprehensively synthesized findings from 35 distinct studies encompassing 1307 patients diagnosed with periodontitis and 1115 healthy control subjects from diverse geographical locations. The evaluation of antioxidant markers in various samples unveiled a nearly equivalent distribution of studies (*n* = 16) focusing on salivary and gingival crevicular fluid (GCF) samples, which did not reveal a prediction pattern. An examination of gingival tissue samples exhibited a predominant trend (*n* = 8) with most studies, except one, demonstrating a compensatory increase in antioxidant enzyme levels at localized tissue sites. In contrast, most analyzed studies (*n* = 15) involving blood and blood‐derived samples portrayed systemic reductions significantly in the status of antioxidants.

### 3.4 Meta‐analysis

The analysis of GSH levels of serum samples revealed a standardized mean difference (SMD) of −5.552 µg/mL (CI 95%: −9.078 to −2.026; *p* − 0.002). On the contrary, the analysis of GPx enzymes in GCF samples displayed an overall SMD of 2.918 ng/µL (CI 95%: 0.372–5.465; *p* − 0.025), and salivary samples exhibited an overall SMD of 0.709 U/l (CI 95%: −1.907–3.325; *p* − 0.596).

**Figure 2:**
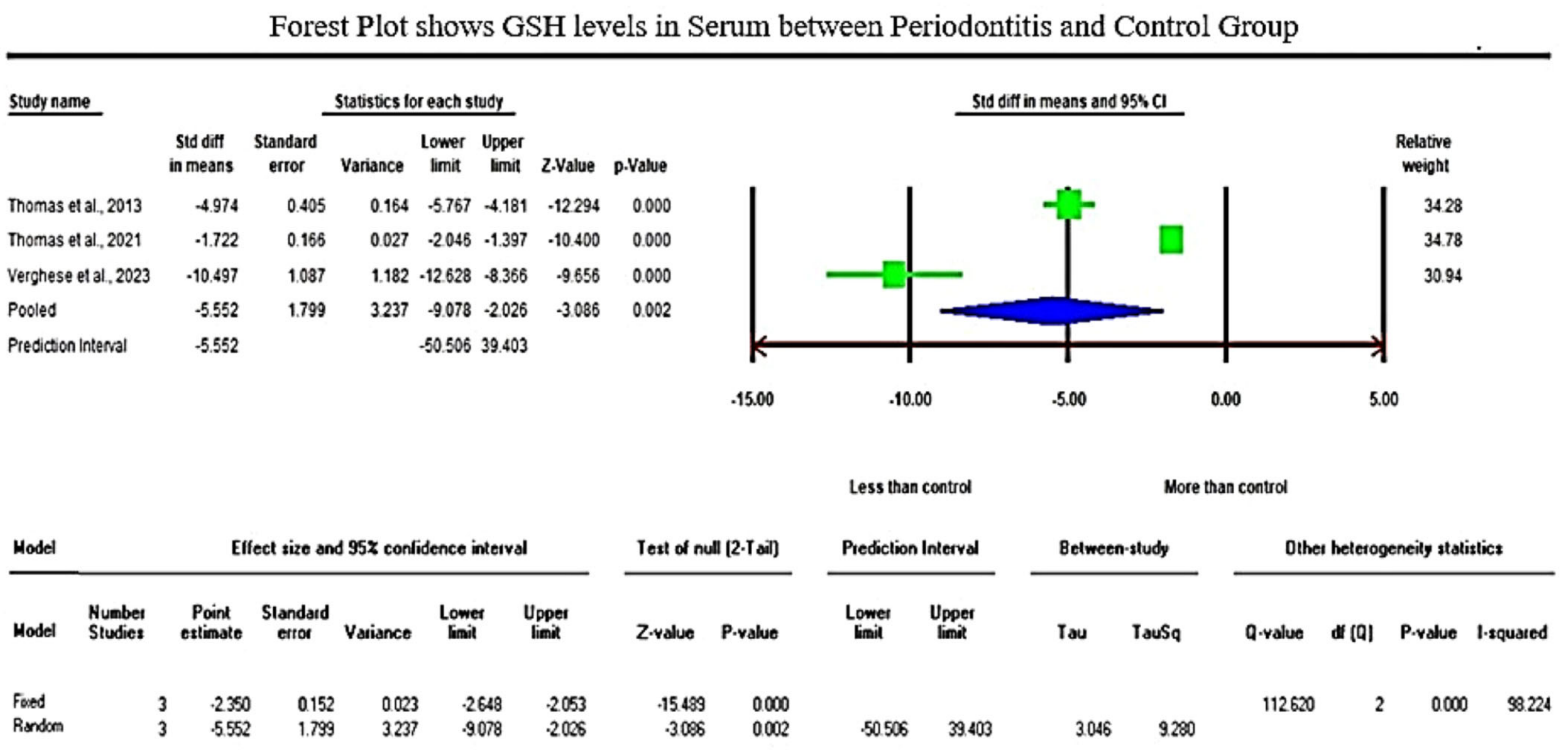
The Forest plot displays standardized mean difference (SMD) values with a confidence interval of 95%, representing the differences in serum glutathione (GSH) levels between patients with periodontitis and the healthy group.

**Figure 3:**
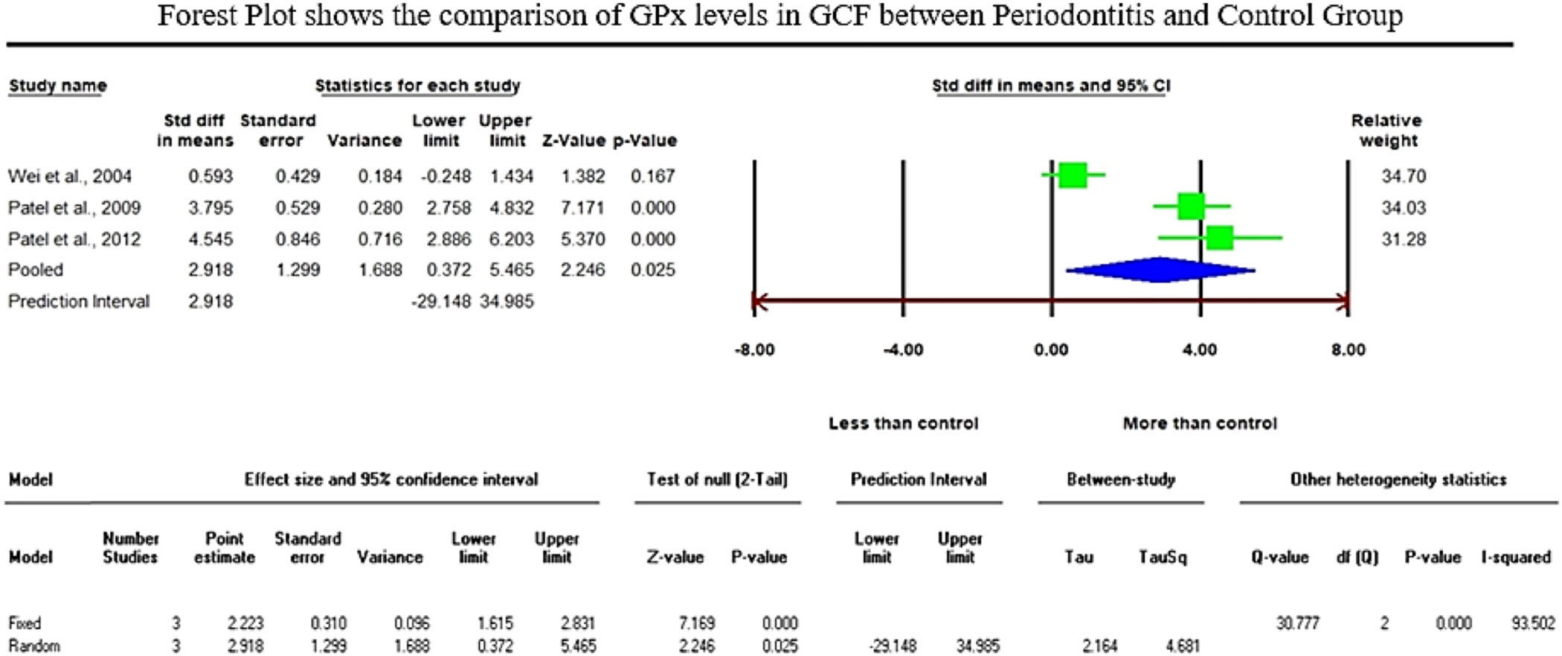
The Forest plot displays standardized mean difference (SMD) values with a confidence interval of 95%, representing the differences in gingival crevicular fluid (GCF), glutathione peroxidase (GPx) levels between patients with periodontitis and the healthy group.

**Figure 4:**
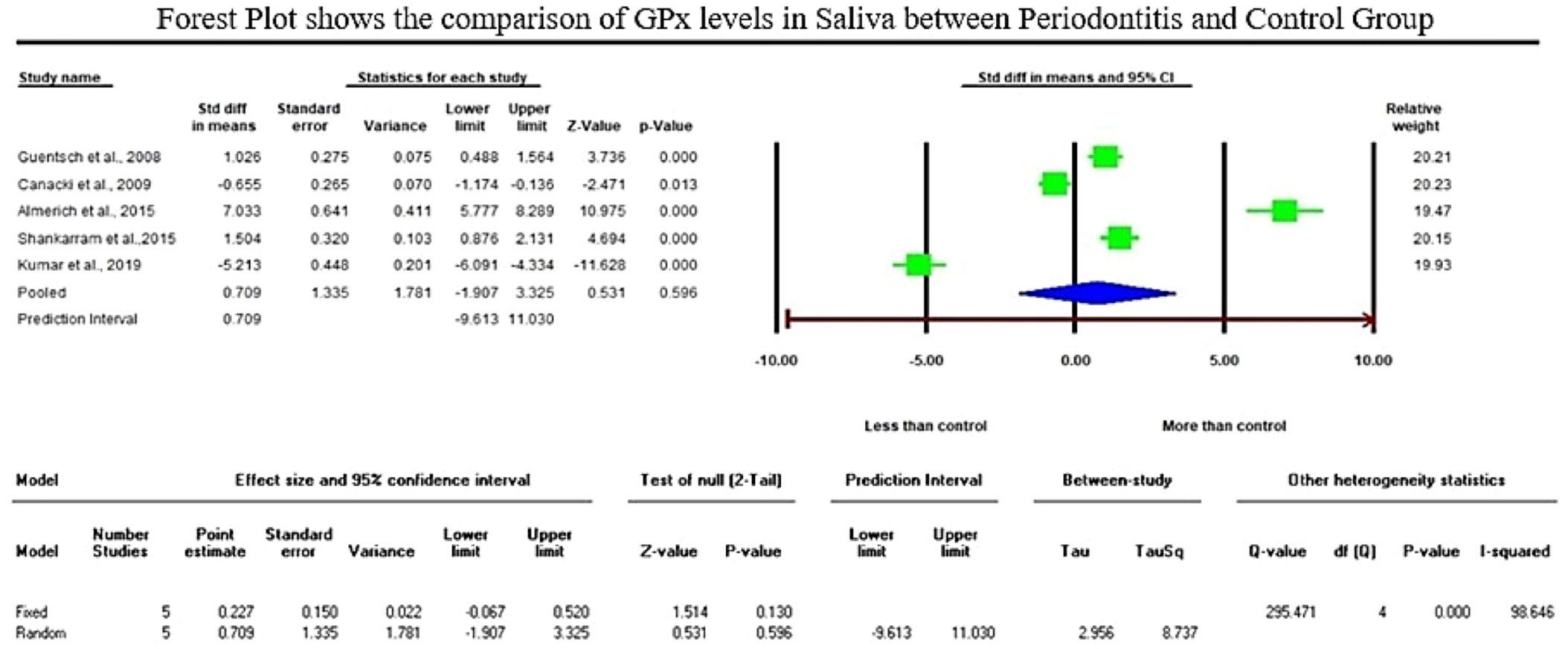
The Forest plot displays standardized mean difference (SMD) values with a confidence interval of 95%, representing the differences in saliva glutathione peroxidase (GPx) levels between patients with periodontitis and the healthy group.

Regarding the meta‐analysis comparing GSH levels between periodontitis and the healthy control group, substantial heterogeneity was observed among the included studies, as indicated by an elevated I² value of 98.224 (Figure 2). The studies involved in the meta‐analysis for GPx values of GCF and saliva displayed significant heterogeneity, represented by I² values of 93.502 and 98.646 in Figures 3 and 4, respectively. This high variability can be attributed to both methodological and biological factors. Variations in measurement protocols followed and differences in the studied population (including sex and age) across different study designs (CS, CC or interventional) contribute to this heterogeneity. The Standardized Mean Difference (SMD) effect scale was employed, and a random effects analysis was utilized to mitigate the discrepancies due to heterogeneity in the meta‐analysis.

Publication Bias: The studies incorporated in the meta‐analysis, comparing the assessment of GSH in serum between the periodontitis and the healthy control group, exhibited Egger's regression intercept of −10.696 with the corresponding two‐tailed probability value of 0.11586. The assessment of GPx levels between periodontitis and the control group revealed the eggers intercept values of 9.872 and 5.112 for GCF and saliva samples, with the corresponding two‐tailed P‐values of 0.42690 and 0.76117, respectively. These findings suggest a reduced likelihood of publication bias in the incorporated studies within the present meta‐analysis.

## 4 DISCUSSION

Over the years, several studies have aimed to unravel the intricate role of antioxidants in periodontitis, mainly focusing on GSH, GR, and GPx due to their potential significance in balancing oxidative stress reaction process. However, the findings across these studies have been somewhat contradictory, adding complexity to the understanding of the role of these enzymes in periodontitis.

When comparing salivary antioxidant enzymes, Trivedi et al. (2014), Trivedi et al. (2015), and Kluknavska et al. (2021) study found a significant decrease in salivary GR activities. Following the previous articles, Tsai et al. (2005) and Senouci et al. (2021) discovered lower GSH concentrations in periodontitis groups compared to control groups. Similarly, six studies noted lower salivary GPx activities in periodontitis patients than in healthy controls (Canakçi et al., 2009; Kluknavská et al., 2020; Kluknavská et al., 2021; Kumar et al., 2019; Miricescu et al., 2014; Novakovic et al., 2013). The reduced antioxidant enzyme leads to an antioxidant defense imbalance, potentially contributing to the inflammatory process observed in periodontitis. Emekli‐Alturfan et al. (2013), Kluknavská et al. (2020), and Kluknavská et al. (2021) studies took a different perspective, reporting increased salivary GSH levels. Kluknavská et al. (2020) study also indicated an increased level of GR in saliva in the periodontitis group compared to the control group. Likewise, six studies reported significantly increased levels of GPx, and Tsai et al. (2005) study displayed a nonsignificant increased level of GPx in the saliva of the periodontitis group compared to the control group (Almerich‐Silla et al., 2015; Guentsch et al., 2008; Hendek et al., 2015; Punj et al., 2017; Shankarram et al., 2015; Smitha et al., 2017). There were only five studies compatible with salivary GPx enzyme meta‐analysis, which exhibited an insignificant rise of GPx in the saliva of the periodontitis group compared to the control group with an overall SMD of 0.709 U/l (95% CI: −1.907–3.325; *p* − 0.596).

When comparing GCF samples, Chapple (2002) and Grant et al. (2010) studies revealed significantly lower GSH and GSSG (reduced and oxidized glutathione) concentrations. Martu et al. (2016) showed a decreased level of GPx in patients with periodontal disease compared to control subjects. On the contrary, five studies reported higher GPx (glutathione peroxidase) levels in GCF from periodontal sites compared to those from normal sites (Hendek et al., 2015; Patel et al., 2009; Patel et al., 2012; Tsai et al., 2005; Wei et al., 2004). The present meta‐analysis comprised three compatible studies for GPx enzyme assessment in GCF samples, which displayed a significant increase of GPx enzymes in the periodontitis group with an overall SMD of 2.918 ng/µL (95% CI: 0.372–5.465; *p* − 0.025). GCF studies provide a unique perspective on the local oxidative stress environment of the periodontal tissues. The collective findings of the varying observations related to antioxidant defense in GCF and saliva could demonstrate the complexity of the antioxidant response within this local microenvironment.

Gingival tissue, a crucial component of the periodontium, is central to the inflammatory processes that characterize periodontitis. Panjamurthy et al. (2005) study reported significantly increased nonenzymatic antioxidant GSH levels. Three studies highlighted an increased GPx (glutathione peroxidase) activity in gingival tissue in the periodontitis group compared to the control group (Panjamurthy et al., 2005; Borges Jr. et al., 2007; Smitha et al., 2017). Borges Jr. et al. (2007) also showed an insignificant increase in GSH levels and reported a significant GSSG and GPx rise in periodontitis sufferers' gingival tissues relative to healthy subjects. This elevation in GSSG, GPx, and GSH could be due to the reflecting intricate response to oxidative stress in the gingival tissue of periodontitis patients. This finding underscores the potential compensatory response of the antioxidant defense system to counteract oxidative stress. However, Borges Jr. et al. (2007) and Tonguc et al. (2011) provided contrasting results by reporting decreased GR and GPx values in the gingival tissue of the periodontitis group compared to the control group, respectively. This discrepancy might be attributed to patient demographics, disease severity, or methodology differences. The conflicting results underscore the complexity of GPx's role in periodontitis and the need for further research in local tissue sites.

While investigating plasma samples, two studies reported a significant decrease in the activities of GR levels, and Panjamurthy et al. (2005) study found significantly lower GSH levels in the periodontitis group when compared to healthy subjects (Gharbi et al., 2019; Kluknavská et al., 2020). Three studies reported reduced GSH levels when analyzing serum samples (Thomas et al., 2013; Thomas et al., 2021; Verghese et al., 2023). The present meta‐analysis of GSH levels of serum samples revealed a significant reduction of GSH levels with a standardized mean difference (SMD) value of −5.552 µg/mL (95% CI: −9.078 to −2.026; *p* −0.002). Similarly, lower serum levels of GPx were reported by five studies in patients with periodontitis when compared to healthy subjects (Dhotre et al., 2012; Aziz et al., 2013; Hendek et al., 2015; Dahiya et al., 2016; Punj et al., 2017). In blood samples, Trivedi et al. (2014) and Sreeram et al. (2015) study reported a significant decrease in GR and GPx activities in periodontitis groups compared to control groups, respectively. Following that, Panjamurthy et al. (2005) and Zuidan (2009) studies reported significantly lower levels of nonenzymatic antioxidants (GSH) in erythrocytes of periodontitis sufferers relative to healthy subjects.

The reduction in GR and GPx activity suggests a potential impairment in the body's ability to regenerate GSH (reduced glutathione), essential for combating oxidative stress, that could compromise the antioxidant defense system. This finding indicates an imbalance in the antioxidant defense system, which could lead to heightened vulnerability to oxidative stress and tissue damage, potentially contributing to the progression of periodontitis. This finding also suggests a systemic impact of periodontitis on the antioxidant defense system, potentially leading to increased oxidative stress throughout the body.

On the contrary, the study by Kluknavská et al. (2020) revealed increased plasma GPx and GSH activity in the periodontitis group compared to the control group, which, following Panjamurthy et al. (2005) study, reported an elevated plasma GPx level. Similarly, another three studies displayed an increase in GPx concentration in erythrocyte, blood, and serum samples among individuals with periodontitis compared to the normal group (Panjamurthy et al., 2005; Tonguç et al., 2011; Patel et al., 2012). The conflicting results underline the intricate nature of the antioxidant response within the bloodstream. Increased antioxidant enzyme activity could be a protective mechanism to neutralize reactive oxygen species and reduce tissue damage.

The observed changes in GR, GSH, and GPx activities in the blood suggest potential disruptions in the redox balance and the body's ability to manage oxidative stress. Collectively, these studies underscore the broad‐reaching impact of periodontitis on antioxidant defense mechanisms, extending beyond the confines of the oral cavity, implicating overall health and potentially contributing to systemic conditions associated with oxidative stress.

## 5 CONCLUSION

The systematic review's findings highlight a significant reduction in antioxidant status across various systemic biological samples in individuals afflicted by periodontitis. This observation contrasts with the outcomes derived from the analysis of gingival tissue samples. Nonetheless, the examination of salivary and gingival crevicular fluid (GCF) samples did not uncover noteworthy differences concerning antioxidant enzyme levels between patients with periodontitis and the control group.

The present findings suggest potential links to systemic conditions associated with oxidative stress. In essence, the research on antioxidant enzymes in the context of periodontitis offers valuable insights into the intricate interplay between oxidative stress and inflammation. The complex and occasionally conflicting outcomes emphasize the necessity for more standardized methodologies and comprehensive investigations to fully comprehend the role of these enzymes in both periodontal health and disease. Further studies are warranted to untangle the underlying mechanisms and explore potential therapeutic avenues for managing conditions related to oxidative stress, such as periodontitis.

## AUTHOR CONTRIBUTIONS

*Conceptualization*: Khadijah Mohideen, Chandrasekaran Krithika, and Snehashish Ghosh. *Methodology*: Snehashish Ghosh and Safal Dhungel. *Software*: Khadijah Mohideen. *Validation*: Khadijah Mohideen and Chandrasekaran Krithika. *Formal analysis*: T. Jeyanthikumari. *Investigation*: N. V. Vani and Khadijah Mohideen. *Resources*: Snehashish Ghosh and Khadijah Mohideen. *Data curation*: N. V. Vani and Khadijah Mohideen. *Writing—original draft formation*: Snehashish Ghosh, Khadijah Mohideen and Chandrasekaran Krithika. *Review and editing*: Snehashish Ghosh and T. Jeyanthikumari. *Visualization and supervision*. Safal Dhungel and Khadijah Mohideen. All authors have approved the completed version of the manuscript.

## CONFLICT OF INTEREST STATEMENT

The authors declare no conflict of interest.

## ETHICS STATEMENT

Given that the current systematic review (SR) and meta‐analysis protocol adheres to PRISMA guidelines and is registered with the PROSPERO registry (CRD42023437112), ethical committee clearance is not necessary.

## Data Availability

Data analyzed in this study are reanalysis of existing data, which are cited in the references.
